# Evaluation of the Effect of Thermal Processing in Total and Bioaccessible Content of Trace Elements in a Regional Crab from the Brazilian Northeast

**DOI:** 10.1021/acsomega.5c04466

**Published:** 2025-08-31

**Authors:** Ana Bárbara Muniz Araújo, Eduardo Mello Barroso Filho, Cleidiane Gomes Lima, Renata Carmo de Assis, Iago Gabriel Medeiros Nobre, Eveline Abreu Menezes, Wladiana Oliveira Matos, Carla Soraya Costa Maia, Francisco Luan Fonsêca da Silva

**Affiliations:** † Laboratório de Análises de Micronutrientes e Alimentos (LAAM), Centro de Ciência da Saúde, 67843 Universidade Estadual Ceará, Campus do Itaperi, Fortaleza, CE 60440-552, Brazil; ‡ 501994 Núcleo de Tecnologia e Qualidade Industrial do Ceará - NUTEC, Fortaleza, CE 60440-552, Brazil; § Laboratório de Estudos em Química Aplicada (LEQA), Departamento de Química Analítica e Físico-Química, 28121 Universidade Federal do Ceará, Campus do Pici, Fortaleza, CE 60455-760, Brazil; ∥ Instituto de Ciências Exatas e da Natureza (ICEN), 245069 Universidade da Integração Internacional da Lusofania Afro-Brasileira, Redenção, CE 62790-000, Brazil

## Abstract

This study evaluated the total and bioaccessible content of essential and potentially toxic elements in crabs of the *Ucides cordatus* species subjected to cooking. In natura and processed samples from different locations in Ceará were analyzed. For this, acid digestion in a microwave oven using 7.0 mL of 65 w w^–1^ HNO_3_ was used for quantification of total elements. A CRM NIST 1568b (Oyster Tissue) was used for trueness evaluation and obtained recoveries between 94 and 103%. An in vitro gastrointestinal digestion method adapted from INFOGEST 2.0 was used to obtain the bioaccessible content of the minerals Al, As, Ca, Cu, Fe, K, Mg, Mn, Se, and Zn, respectively, by ICP OES. The results revealed relevant concentrations of essential minerals in the total contents of Ca (7615 ± 612–17653 ± 716 mg kg ^–1^), Cr (1.77 ± 0.17–6.57 ± 0.27 mg kg ^–1^), Cu (13.2 ± 1.4–87.7 ± 0.5 mg kg ^–1^), Fe (26.18 ± 0.86–53.5 ± 4, 6 mg kg ^–1^), K (5893 ± 260–19932 ± 160 mg kg ^–1^), Mg (1378 ± 21–2359 ± 112.1 mg kg ^–1^), P (3836 ± 18 −4749 ± 249 mg kg ^–1^), Se (0.96 ± 0.04–9.17 ± 0.73 mg kg ^–1^), and Zn (119 ± 8–259 ± 1 mg kg ^–1^), but caution must be done once Cr could be toxic in Cr­(VI) form. Arsenic was detected at levels above the limit allowed by Brazilian legislation, indicating a potential toxicological risk, especially due to the high bioaccessibility (92%) of this element. High bioaccessibility was also observed for Ca (75%), K (100%), Mg (86%), P (96%), and Zn (17%). It can be concluded that *Ucides cordatus* is a significant source of essential minerals, even after heat treatment. However, this food requires constant monitoring for safety due to the presence and high bioaccessibility of potentially toxic elements. These results bring new and innovative information about a traditional food in the Brazilian Northeast, as well as showing how food processing could change the content of trace elements.

## Graphical abstract

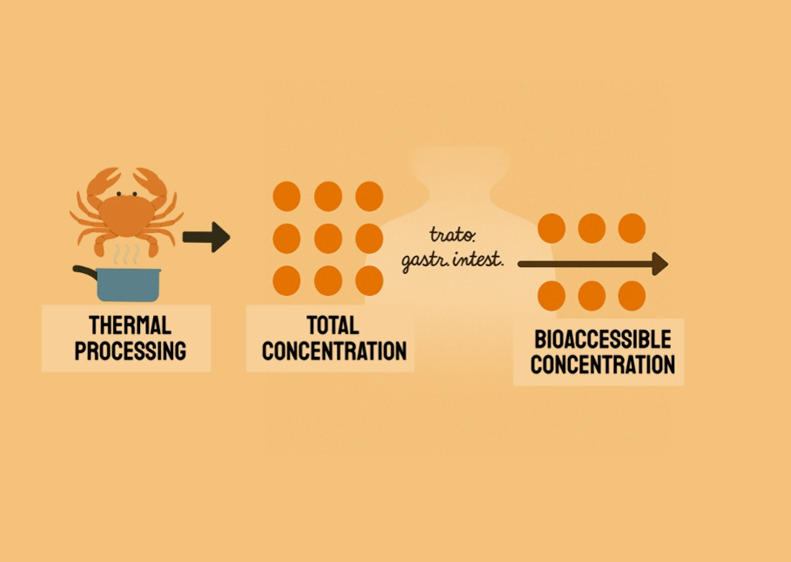

## 1 Introduction

Crab is a crustacean consumed in several cultures, including in Brazil, especially in coastal regions.^1,2^ Among the species found in Brazilian mangroves, especially in the North and Northeast regions, the *Ucides cordatus* (Linnaeus, 1763), popularly known as “uçá crab,” has significant cultural and economic importance, mainly due to its high density in these ecosystems.^1–4^ Furthermore, the species is relatively large and offers a high content of edible parts in the paws and body, which makes it very attractive for direct consumption.

Despite the scarcity of recent official data on the production of the species in Brazil,^5^ production between 2004 and 2007 had an annual average of 9675.^4^ In addition to the economic aspect, the cultural importance of the mangrove crab is highlighted in several Brazilian cities, where it is traditionally consumed in social gatherings, bringing together friends and family in gastronomic events, mainly cooked.^5,6^

Nutritionally, crabs stand out for the significant presence of essential amino acids, fundamental for the growth, repair, and maintenance of body tissues.^7–9^ Additionally, some studies have observed that some species, such as *Scylla paramamosain*, *Eriocheir sinensis*, and *Scylla serrata*, provide important minerals, such as iron (Fe), calcium (Ca), magnesium (Mg), phosphorus (P), zinc (Zn), and selenium (Se), essential for bone health, immune function and energy production.^10–12^

In the Brazilian Food Composition Table (TBCA), specifically for the mangrove crab (*Ucides cordatus*), average values of 2.10 mg for Fe, 356 mg for Ca, 52.2 mg for Mg, 153 mg for P, and 6.14 mg for Zn per 100 g are reported, while selenium was not detected.^13^

Despite these benefits, crab consumption may represent potential exposure to contaminants, such as potentially toxic elements, especially in riverine and fishing communities.^7,14^ Crustaceans live in direct contact with benthic sediments, absorbing these elements from the environment through their gills.^15^ Although many trace elements are naturally found in the marine environment, a significant portion comes from human activities, resulting in the presence of potentially toxic elements such as arsenic (As), cadmium (Cd), cobalt (Co), nickel (Ni), mercury (Hg), and lead (Pb), which are often associated with organic particles suspended in water and sediments, facilitating their bioaccumulation in crab tissues and, consequently, throughout the food chain.^15^

Several studies have investigated both the nutritional aspects and the risks related to the presence of contaminants in crabs. Some of these have highlighted the nutritional benefits associated with crab consumption, evidencing its role as a relevant source of essential nutrients.^11,16–20^ On the other hand, many studies focus on the occurrence of potentially toxic trace elements in these crustaceans.^21–26^ In this context, Brazilian legislation establishes the maximum tolerated limits for specific contaminants in crustaceans, such as arsenic, cadmium, lead, chromium, and copper.^27^

However, for these elements to exert their beneficial or toxic effects on the body, it is essential to consider the bioavailable levels, which correspond to the fraction available for biological utilization by cell metabolism.^28,29^

Nevertheless, bioavailability tests have several limitations, especially those related to cost and ethical principles, so methods that simulate in vitro digestion have been developed to estimate the bioaccessible content of food. This analysis does not assess how much of the nutrient is absorbed by the organism, but only the fraction of minerals soluble and available for absorption.^29^ However, the in vitro technique has some advantages over in vivo studies, such as good control of variables and low cost.

The development of methods for determining total and bioaccessible levels is still relevant due to the wide variety of samples and the number of regional and traditional foods that require this information.^30,31^ The ICP OES is a versatile multielemental analytical technique ideal for the analysis of macro and micronutrients in foods and their bioaccessible fractions due to its wide linear range, selectivity, sensitivity, and precision, being widely used for the analysis of trace elements in several works in the literature.^30–32^

One of the important variables in bioaccessibility analysis is thermal processing, which causes changes in the physical and chemical structure of food matrices, affecting the content of bioaccessible minerals, as well as inserting antinutritional compounds that could decrease the bioavailability of trace elements.^33,34^

By promoting an understanding of the effectively absorbable fraction of elements present in crabs, this research can support the formulation of public policies aimed at reducing environmental impacts and preventing diseases due to both exposure to potentially toxic elements and nutritional deficiencies from minerals.^35^

Thus, bioaccessibility studies are low-cost experimental tools with greater control over variables, allowing for estimation of the available mineral and contaminant content in a sample. Even though the bioaccessible, or soluble, value does not yet represent the content effectively absorbed at the cellular level, it is a reliable estimate and less costly than in vivo or cell culture experiments.

This study aimed to quantify the total trace element content and bioaccessibility fraction in samples of regional crabs, assessing the influence of thermal processing on the total and bioaccessible levels of these elements. Furthermore, there are few studies on the inorganic characterization and bioaccessible content of the studied crab species; therefore, these data are extremely important for assessing the food safety of traditional food, bringing new data to the literature.

## 2 Materials and Methods

### 2.1 Samples

Crabs of the species *Ucides cordatus* were purchased in local market from three different local shops in the state of Ceará: approximately 1 kg of fresh sample (approximately 10 crabs) was purchased in mucipality of Eusebio (3° 53′ 24″ S, 38° 27′ 03″) and Amontada (3°21′39″ S, 39°49′51″) in state of Ceará on August of 2024. In addition to the location, the samples differ in the method used to sacrifice the animal, which can be by precooking in boiling water (1–2 min) (Amontada’s sample) or in an ice bath (Eusebio’s sample). A sample of industrialized crab meat was purchased from a traditional seafood market and corresponds to the edible part of the animal that is removed after precooking and stored for sale.

The in natura samples, which had already been sacrificed, were initially washed with ultrapure water, and the carapace and legs were manually separated to standardize the edible portion analyzed. The three samples were then boiled under ultrapure water in a stainless-steel pan for 20 min at approximately 180 °C.

After heat treatment, all three samples were freeze-dried and then ground in a food processor with stainless-steel blades by using a sacrificial sample to minimize the risk of contamination.

To assess the trueness of the analytical method, the analysis of certified reference material was carried out using SRM 1566b *Oyster Tissue* (NIST, EUA).

### 2.2 Instrumentation

The samples were decomposed using a closed microwave-oven system with a Start D cavity (Milestone), equipped with 12 Teflon flasks with a capacity of 50 mL.

The materials used in the kitchen for heat treatment were stainless-steel pans, ceramic, and polyethylene instruments to avoid contamination.

For in vitro gastrointestinal digestion, a Dubnoff thermostatic bath with agitation was used to incubate the samples.

The trace elements were determined using an iCap 6000 inductively coupled plasma optical emission spectrometer (ICP OES) (Thermo Scientific), equipped with a concentric nebulizer and a cyclonic nebulization chamber. The ICP OES operating parameters were as follows: radio frequency power of 1.55 kW; argon plasma flow of 15 L min^–1^; auxiliary argon flow of 0.6 L min^–1^; carrier argon flow of 1.02 L min^–1^; and sample flow of 1.4 L min^–1^. The elements were measured using axial vision at the following wavelengths: 308.2 nm (Al), 193.8 nm (As), 422.3 nm (Ca), 228.8 (Cd), 267.7 nm (Cr), 327.4 nm (Cu), 259.9 (Fe), 766.5 nm (K), 285.2 nm (Mg), 257.6 nm (Mn), 177.5 nm (P), 630.0 nm (Pb), 196.1 nm (Se), and 206.2 nm (Zn).

### 2.3 Reagents and Solution

Solutions were prepared using ultrapure water (18.2 MΩ cm resistivity) obtained from a Milli-Q water purification system (Millipore, Bedford, MA, USA). All glassware was immersed in 10% v v^–1^ HNO_3_ (Sigma-Aldrich, Germany) for 24 h and thoroughly rinsed with ultrapure water. The extraction procedures and digestions were accomplished using 65% w w^–1^ HNO_3_ (Sigma-Aldrich).

Nitric acid (HNO_3_) 65% w w^–1^ (Vetec, Rio de Janeiro) was used for the sample preparation procedures. The digestive fluids for simulating the in vitro test were prepared using the following reagents and enzymes: 98% w w^–1^ sodium bicarbonate (NaHCO_3_), 37% w w^–1^ hydrochloric acid (HCl), >98% w w^–1^ sodium hydroxide (NaOH) (Sigma-Aldrich, Germany), porcine gastric mucosal pepsin P7000 (≥250 units/mg), porcine pancreatin P1750 and bile salts (≈50% w w^–1^ sodium cholate and 50% w w^–1^ sodium deoxycholate) (Sigma-Aldrich).

The analytical curves for quantifications by ICP OES were prepared from successive dilutions of 1000 mg L^–1^ Specsol (São Paulo, Brazil) standard solutions in the range between 1.0 and 50 mg L^–1^.

### 2.4 Total Decomposition

The crab samples were wet digested in triplicate. Approximately, 0.500 g of each sample was weighed into Teflon tubes, and 7 mL of 65% w w^–1^ HNO_3_ was added. The tubes were heated in a microwave oven using the following heating ramp: step 1:100–600 W for 5 min; step 2:600 W for 5 min; step 3:600–1000 W for 0 min; step 4:1000 W for 10 min; step 5:0 W for 15 min. After cooling the flasks to room temperature, the samples were diluted to 25 mL with ultrapure water and carried for analysis by ICP OES. Certified reference material was decomposed using the same conditions to assess the veracity of the decomposition.

### 2.5 In Vitro Gastrointestinal Digestion

In vitro digestion for bioaccessibility analysis was carried out using the methodology of Menezes and collaborators.^34^

To do this, 0.500 g of the samples and 10 mL of ultrapure water were added to a 50 mL Falcon flask. After 15 min, 0.25 mL of gastric solution (10% w v^–1^ pepsin in 0.1 mol L^–1^ HCl) was added, and the pH was adjusted to 2.5 with 0.01 mol L^–1^ HCl, and then, the samples were placed in a thermostatic bath at 37 °C and 150 rpm for 2 h. After this, the samples were placed in an ice bath for 10 min to stop the enzymatic activity. In the intestinal stage, 2.5 mL of intestinal solution was added (0.2% w v^–1^ pancreatin and 1.25% w v^–1^ bile salts in a 0.1 mol L^–1^ NaHCO_3_ medium) and the pH was adjusted to 7.4 with a 0.01 mol L^–1^ NaOH solution. Once again, the flasks were placed in a thermostatic bath at 37 °C for 2 h, stirring at 150 rpm. Finally, the sample was centrifuged, and the analysis of the supernatant was considered the bioaccessible fraction.

The following eq 1 was used to determine the bioaccessibility of each element.

%BA=(BF/TF)×100 1

where BA is the percentage of bioaccessibility, BF is the bioaccessible fraction (mg kg^–1^), and TF is the total mass fraction (mg kg^–1^).

### 2.6 Statistical Treatment

For statistical analysis of the similarity between the concentrations of the analytes obtained between the samples, the ANOVA test and Tukey’s test were carried out in the JASP Team software year 2025 (version 0.19.3). Statistical analysis to determine the difference in analyte concentrations between processing was tested using Student’s *t*-test for equal or different variances, which was verified using the F-test. A significant level of 5% was assumed for each analysis.

To analyze the limits of detection and quantification, the analysis of 10 independent blanks was used using the equations below:

LOD=3×RSD×BEC 2

LOQ=10×RSD×BEC 3

$$\mathrm{BEC}=I_{\mathrm{blank}}/s$$ 4

where RSD is the relative standard deviation of 10 independent blanks, *I*_blank_ is the intensity of emission of the blank sample, and *s* is the slope of the analytical curve obtained.

## 3 Results and Discussion

### 3.1 Some Analytical Considerations

To assess the trueness of the results of the analysis of the total mineral content, samples of SRM 1566b Oyster Tissue were analyzed using the same experimental procedure applied to the analysis of the total mineral content in the crab sample in dry mass.

This certified reference material (CRM) was chosen because its matrix is similar to crustaceans, as it is recommended that the CRM be similar to the analyzed sample. Furthermore, the same CRM has already been used in literature to validate methods for trace element analysis in other crustaceans.^36^

The results obtained for the total concentration of trace elements in crab samples are shown in Table 1.

**Table 1:** Total Contents of Al, As, Ca, Cu, Fe, K, Mg, Mn, Se, and Zn in SRM NIST 1566b Oyster Tissue Sample Determined by ICP OES after Microwave-Assisted Digestion (Mean ± SD, *n* = 3)

	NIST SRM 1566b: oyster tissue		
elements (mg kg^–1^)	MW digestion	certified material^a^	% recovery
Al	198.5 ± 10.2	197.2 ± 6.0	100
As	7.16 ± 0.80	7.65 ± 0.08	94
Ca	850 ± 62	838 ± 20	101
Cu	70.8 ± 6.2	71.6 ± 0.8	99
Fe	207.7 ± 15.3	205.8 ± 3.4	101
K	6625 ± 16	6520 ± 90	102
Mg	1105 ± 10	1085 ± 23	102
Mn	18.2 ± 0.5	18.5 ± 0.1	98
Se	2.12 ± 0.15	2.06 ± 0.07	103
Zn	1418 ± 18	1424 ± 46	99

a Mean ± U, *k* = 2.

According to the data in Table 1, all the element contents found in the SRM NIST 1566b *Oyster Tissue* sample had satisfactory analytical recoveries (94–103%) with concentrations statistically similar to the reference value (Student’s *t* test, 95% confidence), which supports the trueness of the total mineral content analyses in the crab samples.

The LOD and LOQ for the present method are described in Table 2.

**Table 2:** LOD and LOQ for Al, As, Ca, Cd, Cr, Cu, Fe, K, Mg, Mn, P, Se, and Zn Determination in the Crab Sample after MW Digestion and Analysis by ICP-OES

elements	LOD (mg L^–1^)	LOQ (mg L^–1^)
Al	0.04	0.13
As	0.06	0.20
Cd	0.05	0.17
Cr	0.01	0.33
Cu	0.17	0.59
Fe	0.07	0.25
K	0.01	0.03
Mg	0.01	0.03
Mn	0.03	0.10
P	0.02	0.07
Pb	0.30	1.00
Se	0.10	0.35
Zn	0.05	0.17

The detection and quantification limit data show the ability of the analytical method to meet the limits of national legislation and international recommendations.

### 3.2 Total Content

The total concentrations of the elements Al, As, Ca, Cr, Cu, Fe, K, Mg, Mn, P, Se, and Zn in the crab samples are shown in Table 3, together with the percentage loss during cooking (%PM).

**Table 3:** Determination of Total Al, As, Ca, Cr, Cu, Fe, K, Mg, Mn, P, Se, and Zn (mg kg^–1^) in Raw and Cooked Crab (*Ucides cordatus*) Samples after Decomposition by MW and Analysis by ICP OES (Mean ± SD, *n* = 3)^a^

	sample 1			sample 2			industrialized crab meat		
elements (mg kg^–1^)	in natura	cooked	PM%	in natura	cooked	PM%	in natura	cooked	PM%
Al	210 ± 19^a^	227 ± 16^a^	0	361 ± 9^b^	223 ± 5^a^	38	235 ± 4^a^	114 ± 16^c^	47
As	1.52 ± 0.12^a^	1.14 ± 0.06^b^	25	0.43 ± 0.01^a^	0.49 ± 0.06^a^	0	0.90 ± 0.10^a^	0.76 ± 0.02^a^	16
Ca	9905 ± 257.7^a^	7615 ± 612^b^	23	12363 ± 406^a^	12646 ± 1155^a^	0	17653 ± 716^c^	12048 ± 881^a^	32
Cr	3.76 ± 0.35^a^	4.06 ± 0.27^a^	0	6.57 ± 0.27^b^	4.03 ± 0.00^a^	39	3.60 ± 0.08^a^	1.77 ± 0.17 ^c^	50
Cu	53.1 ± 0.8^a^	52.2 ± 1.4^a^	2	87.7 ± 0.5^b^	87.3 ± 0.63^b^	0.5	18.1 ± 0.42^c^	13.2 ± 1.4^d^	27
Fe	29.6 ± 0.73^a^	26.18 ± 0.86^a^	0	43.2 ± 0.72^b^	26.8 ± 0.21^a^	38	53.5 ± 4.6^b^	52.6 ± 2.6^b^	1,7
K	19932 ± 160^a^	9414 ± 108^b^	53	7452 ± 670^c^	5893 ± 260^d^	21	17733 ± 313^a^	7046 ± 202^c^	60
Mg	1537 ± 42^a^	1378 ± 21^a^	10	2253 ± 34 ^b^	1462 ± 72^a^	35	2359 ± 112.1^b^	1563 ± 129^a^	34
Mn	1.20 ± 0.04^a^	1.18 ± 0.1^a^	2	2.70 ± 0.19^b^	2.00 ± 0.20^c^	25	4.70 ± 0.50^d^	3.20 ± 0.10^b^	31
P	4538 ± 75^a^	4458 ± 140^a^	0	3836 ± 18^b^	4515 ± 168^a^	0	4735 ± 85^a^	4749 ± 249^a^	0
Se	3.34 ± 0.093^a^	2.96 ± 0.32^a^	11	9.17 ± 0.73^b^	6.60 ± 0.67^c^	28	1.4 ± 0.07^d^	0.96 ± 0.04^e^	30
Zn	232 ± 5^a^	220 ± 17^a^	5	259 ± 5^a^	241 ± 1^a^	7	175 ± 1^b^	119 ± 8^b^	32

a Equal letters on the same line represent statistically similar values (95% confidence level). %PM percentage of mineral loss. When statistically similar, %PM was considered 0.

Crab consumption can represent an important source of nutrients and, at the same time, potential exposure to toxic elements.^7,14,26^ Despite their significant contribution to the intake of essential minerals, most studies focus on determining potentially toxic elements, such as As, Cd, Hg, and Pb, and the risks associated with their ingestion through the consumption of crabs and other crustaceans.^21–26^ These elements can accumulate along the aquatic food chain, posing potential risks to marine organisms and, consequently, to human food safety.^37^ When present in concentrations exceeding the organism’s detoxification capacity, these elements can cause deleterious effects on important biological functions: As is associated with carcinogenicity;^38^ Cd induces oxidative stress;^39^ and Pb affects multiple organs, presenting limited elimination capacity by the body.^40^ Potential risks to marine organisms include environmental impacts, ecotoxicological effects, and bioaccumulation^41^

In this study, the concentrations of Cd and Pb were lower than the detection limit of the technique (0.05 mg kg^–1^), which brings them within the current Brazilian legislation of 0.50 mg kg^–1^,^27^ while the Hg content was not assessed.

The concentration of As observed in sample 1 exceeded the maximum tolerated limit for total As in crustaceans, according to Brazilian Legislation (1 mg kg^–1^),^27^ which is a worrying finding given its toxic potential.^36^ This element is commonly found in seafood, mainly in the form of arsenobetaine, which is considered nontoxic.^42^ Although this study did not differentiate the chemical specification of As, literature points to the predominance of organic forms in fish and seafood, while inorganic As, which is more toxic, tends to be less frequent.^42,43^ In a study that analyzed the speciation of As in a sample of the *Eriocheir sinensis* species in China, the most dominant form was arsenobetaine.^44^

Comparing the results obtained with data from the literature, it was observed that the arsenic levels found in this study were higher than those reported by Tavares, Brito, and Kujbida,^45^ who found an average of 0.660 mg kg^–1^ of As in crab meat purchased from different commercial outlets in the city of Natal/RN, also in northeastern Brazil. However, in an analysis conducted with the species *Callinectes sapidus*, collected in the Niger Delta region of Nigeria, higher concentrations of arsenic were reported, ranging from 1.77 to 6.03 mg kg^–1^. exceeding the limit set by the USEPA of 1.20 mg kg^–1^.^46^ Analyses of other crustaceans from the same region as the crab samples also showed arsenic values much higher than those permitted in the literature, particularly shrimp samples, which had As levels between 4 and 11 times higher than those permitted by legislation.^42,43^

Another element that stands out is Al; the concentrations in this study are higher than those reported in crabs of the *Cardisoma armatum* species in the coastal town of Kribi, in the Republic of Cameroon.^37,47^ Exposure to high concentrations of this element is associated with health risks, including neurodegenerative disorders and cell damage due to its accumulative effect.^48^ High concentrations of Al have also been reported in seaweed samples from the coast of Ceará. Algae are also important biomarkers, and these data may corroborate the increase in the level of this element caused by human action, especially due to the installation of a steel mill in the coastal region of the state.^49^

As environmental bioindicators, crabs accumulate these elements due to their direct exposure to benthic segments, which are the main reservoirs of metals in the aquatic environment.^14^ In addition to the risks associated with contamination, benthic sediments also act as a reservoir of essential minerals, such as Fe, Mg, and Zn,^50^ which may justify the high concentrations of these elements in the samples analyzed (Table 3), corroborating the findings in the literature.

It is important to note that there is great variability in the trace element content of the different samples, which indicates that the presence/absence of the shell, as well as the mode of sacrifice (hot water or ice bath), can suggest significant changes in the trace element content.

In addition, the cooking process shows changes in the trace element profile of the samples under study (Table 3).

Table 3 shows that in crab sample 1, cooking led to significant reductions in the levels of As (25%), Ca (23%), and K (53%). In sample 2, there was a reduction in Al (38%), Cr (39%), Fe (38%), K (21%), Mg (35%), Mn (25%), and Se (28%). In the industrialized crab meat, there was a significant reduction in the elements Al (37%), Ca (32%), Cr (50%), Cu (27%), K (60%), Mg (34%), Mn (31%), and Se (30%). These reductions can be attributed to the leaching of elements into the liquid medium during cooking.^33,34^

It is important to note that the industrialized crab meat, which went through the boiling process without the shell, behaved differently from the crab that was cooked whole. This difference can be explained by the absence of structures such as the shell and legs during thermal processing, parts of the crab that concentrate minerals such as Ca and Mg, fundamental elements for hardening the shell.^51^ This shell may have acted as a physical barrier, significantly reducing the loss of minerals by leaching during the cooking of the crab. This also shows the difference observed in the crabs sacrificed in boiling water (sample 2), which had more losses than the crabs in the ice bath (sample 1), most likely due to losses in this water, even if only for a short time.

It is important to note that there was a reduction in the As content only in sample 1, and even in sample 2, which was cooked with the shell, there was no reduction. This may be related to the ability of organic species of As to be present in the structure of animal muscle tissue and thus less likely to be leached^42,52^

Another relevant point is the significant reduction in Al content in samples 2 and 3 after cooking. It is worth noting that, in cooking, it is common to reuse the cooking water from various foods in preparations such as broths, soups, and sauces. Therefore, the water resulting from cooking the crab could also be used, thus maintaining potential exposure to the elements leached by the water.

It is worth noting that the presence of nutritionally relevant elements, considering the role of Ca in bone development and maintenance, and consequently in the prevention of osteoporosis;^53^ Se plays a fundamental role in cellular antioxidant defense and in maintaining redox homeostasis;^54^ Zn is essential for immune function, since its homeostasis determines anti-inflammatory and redox reactions, as well as the recovery of the number of immune cells;^37^ and K is associated with osmotic regulation and the reduction of cardiovascular risks.^37^

In general, the minerals studied, even after the losses, maintained relevant levels and play an important role in maintaining the normal function of the immune system, as highlighted in the literature.^55^

### 3.3 Bioaccessible Fraction

The total concentration of the elements quantified in the samples does not represent the real content of elements available for intestinal absorption, but rather the bioaccessible fraction. The bioaccessible contents are listed in Table 4, while the percentage of bioaccessibility (%BA) is illustrated in Figure 1.

**Table 4:** Bioacessbile Content (mg kg^–1^, Mean ± SD, *n* = 3)

	sample 1		sample 2		industrialized crab meat	
elements (mg kg^–1^)	in natura	cooked	in natura	cooked	in natura	cooked
Al	2.34 ± 0.02	0.91 ± 0.02	<LOQ	<LOQ	0.168 ± 0.02	<LOQ
As	1.35 ± 0.09	0.75 ± 0.18	0.27 ± 0.01	0.45 ± 0.05	0.43 ± 0.01	0.81 ± 0.08
Ca	1334 ± 135	5738 ± 458	6155 ± 509	5045 ± 312	6689 ± 642	4274 ± 176
Cr	0.23 ± 0.01	0.11 ± 0.01	0.12 ± 0.02	<LOQ	0.14 ± 0.01	<LOQ
Cu	6.42 ± 0.06	4.75 ± 0.07	5.86 ± 1.41	1.18 ± 0.05	13.2 ± 0.8	8.20 ± 0.69
Fe	0.19 ± 0.01	0.05 ± 0.08	0.33 ± 0.03	0.07 ± 0.01	4.30 ± 0.24	5.04 ± 0.05
K	15085 ± 733	9720 ± 452	7536 ± 199	6576 ± 378	897 ± 34	399 ± 5
Mg	108 ± 10	1188 ± 124	2558 ± 247	201 ± 12	694 ± 50	856 ± 39
Mn	<LOQ	0.156 ± 0.01	0.10 ± 0.01	0.12 ± 0.01	0.21 ± 0.02	0.63 ± 0.10
P	533 ± 29	4292 ± 373	151 ± 8	2074 ± 27	2347 ± 18	1965 ± 128
Se	3.35 ± 0.16	1.90 ± 0.01	1.06 ± 0.11	1.89 ± 0.02	0.87 ± 0.09	0.43 ± 0.04
Zn	77.7 ± 5.3	49.5 ± 7.2	17.6 ± 0.12	14.0 ± 0.91	33.4 ± 1.5	19.8 ± 0.7

**Figure 1:**
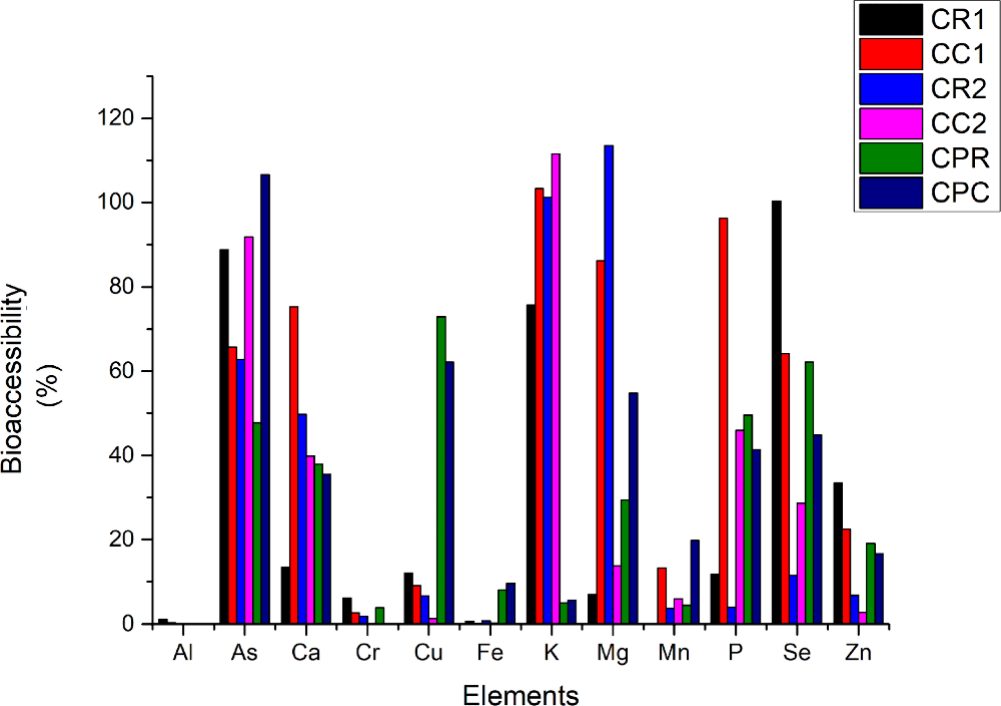
Percentage bioaccessibility of minerals before and after cooking crab samples CR1 and CR2 are the Crab in natura, CC1 and CC2 are the crabs cooked, CPR are the Industrialized crab meat in natura, and CPC the industrialized crab meat cooked.

Figure 1 shows the percentage of bioaccessibility for the elements in study.

Considerable bioaccessibility percentages were observed for the elements Ca (75%), K (100%), Mg (86%), and P (96%) in sample 1, while in sample 2, the predominant elements were As (92%) and K (100%). In the industrialized crab meat sample, high bioaccessibility was found for As (100%), Mn (20%), and Zn (17%).

The high %BA of As, both in the raw state and after cooking, reinforces concern about its potential toxic effect. These data indicate that, even after heat treatment and simulated digestion, arsenic remains available for absorption in the gastrointestinal tract, highlighting the need for a more detailed assessment of its chemical forms.^42^ High levels of bioaccessible concentrations of As in another crustacean were observed even after cooking.^56^

Another relevant aspect is the variation in the aluminum content. Although the total content of this element is high compared to other crab species,^37,47^ the low %BA observed suggests limited availability for absorption in the human organism, most likely due to the low Kps values for Al­(OH)_3_ (5.0 × 10^–33^), which causes the element to be precipitated and excreted. Furthermore, even though only a small fraction of this element is available for absorption, there is a danger of absorption of this elemen,t which is linked to various neurological diseases such as Parkinson’s and Alzheimer’s.^46^

As can be seen in Figure 1, the cooking process generally increases the percentage of bioaccessibility for most of the elements studied. This same trend has been observed in various studies assessing bioaccessibility in animal proteins such as liver, beef, chicken, fish, and shrimp.^33,34,52^ As well as increasing protein digestibility, temperature can transform some organic structures linked to minerals, such as arsenic and selenium compounds.

However, an opposite effect is observed for Zn, where there is a decrease in the bioaccessibility after cooking. This may be related to the denaturation of proteins induced by heating, which alters the conformation of the food matrix and can promote the release of zinc from soluble complexes, favoring the formation of precipitates, which reduces its absorbable fraction.^54^ A similar effect was observed in another study, in which cooking in water also resulted in a decrease in the bioaccessibility of zinc.^33^

### 3.4 Nutritional Contribution

In addition to its cultural and economic value, crab stands out for its high nutritional value, being an excellent source of protein of high biological value, essential for the growth, maintenance, and repair of body tissues.^8,9^ Several studies show that seafood, including crabs, is lower in saturated fat compared to red meat.^12^

It is therefore important to understand the nutritional contribution of micronutrients from crab consumption. In addition, food composition tables only show the total content of this element, and often, regional foods are not described or are described with outdated data.

One way of understanding the contribution of food to the intake of a nutrient is through the concept of Recommended Daily Allowance (RDA),^57^ which is shown in the Supporting Information (S1).

Considering that a kilogram of large crab corresponds, on average, to around 6–7 units, with a loss of approximately 15–20% of the weight after cooking, it is estimated that 100 g of cooked crab is equivalent to approximately 1 unit. This consumption can partially or totally meet the Ca RDA for adult men aged 31–50 (S1). The high concentration of Ca suggests that crab can serve as an alternative in diets free from natural sources of Ca, such as dairy products.

About K, its concentration remained high in crab 1 and crab 2 samples, even after simulated digestion. This result becomes relevant when analyzed in accordance Brazilian regulation,^58^ which defines as a “source” food that provide at least 15% of the RDA and with the Federal Food, Drug, and Cosmetic Act of the Food and Drug Administration (FDA), which classifies as a “good source” food that contribute 10% to 19% of the RDA. In this context, thermal processing has proved to be advantageous, possibly because it does not involve industrial steps that favor the loss of this mineral.

When we look at P, in addition to its high bioaccessibility, it remained significant even after in vitro digestion in the samples analyzed. The same was observed for Mg, whose concentration was also classified as rich (>15% of the RDA) in all the samples. These findings reinforce the nutritional relevance of the mangrove crab, considering that phosphorus is essential for the formation and maintenance of bones and teeth, as well as acting in the regulation of energy metabolism, while magnesium participates in various enzymatic reactions, contributes to neuromuscular function, and is involved in protein synthesis.^59^

The concentrations of bioaccessible Se in the samples are noteworthy, since this element is essential and may be related to a natural increase in this element in the north and northeast of the country.^54^ Selenium exerts biological activity through selenoproteins, which are responsible for controlling thyroid hormone, fertility, aging, and immunity, and play a fundamental role in maintaining redox balance in cells.^59^ Their absorption, transport, distribution, and reuse can sometimes be improved when inserted into a food matrix.^60^

Although not all of the elements essential to the body are present in the samples analyzed, it can be seen that the samples are sources of Ca, Cr, Cu, K, Mg, P, Se, and Zn. In this way, they can be included in the human diet, especially when considering the inclusion of regional food and the use of local ingredients in food to perpetuate a gastronomic culture.

However, it is important to note that this study shows the incidence of high values of As and Al in the samples that remain after cooking, or are leached into the water, which can be a problem in the consumption of broths, soups, and preparations that use this cooking water; in addition, many elements are found in high quantities, and excessive consumption can cause problems related to contamination, or high intake of micronutrients.

## 4 Conclusions

It can be concluded that *Ucides cordatus* has nutritional relevance with higher bioaccessible levels of essential trace elements as Ca (75%), K (100%), Mg (86%), P (96%), Mn (20%), and Zn (17%) in the cooked sample. However, its safety for human consumption requires attention, especially in relation to arsenic and aluminum content.

This work provides important information on the characterization of a native species and its nutritional importance, as well as showing how thermal processing and the way in which the food is sacrificed and stored can modify its inorganic content. However, more studies using cell models are required to understand the bioavailability of these nutrients and toxic elements.

## Supplementary Material



## Notes

Data relating total and bioaccessible levels to recommended dietary allowance (RDA) values (PDF)
