# Supplementary material for: Evaluation of the Effect of Thermal Processing in Total and Bioaccessible Content of Trace Elements in a Regional Crab from the Brazilian Northeast
Source: ACS Omega. 2025 Aug 31;10(36):41386–94. doi: 10.1021/acsomega.5c04466 (PMC12444536; doi:10.1021/acsomega.5c04466)
Supplement: Supplementary file 1 [file ao5c04466_si_001.pdf]

Evaluation of the effect of thermal processing in total and bioaccessible content of trace elements in a regional crab from the brazilian northeast

Ana Bárbara Muniz Araújo[a], Eduardo Mello Barroso Filho[a], Cleidiane Gomes Lima[b], Renata Carmo de Assis[a], Iago Gabriel Medeiros Nobre[c], Eveline Abreu Menezes [d], Wladiana Oliveira Matos[c], Carla Soraya Costa Maia[a], Francisco Luan Fonsêca da Silva[a*]

[a] Laboratório de Análises de Micronutrientes e Alimentos (LAAM), Centro de Ciência da Saúde, Universidade Estadual Ceará, Campus do Itaperi, Fortaleza, Ceará, Brazil, 60440-552

[b] Núcleo de Tecnologia e Qualidade Industrial do Ceará - NUTEC, Fortaleza, CE, Brazil, 60440-552,

[c]Laboratório de Estudos em Química Aplicada (LEQA), Departamento de Química Analítica e Físico-Química, Universidade Federal do Ceará, Campus do Pici, Fortaleza, CE, Brazil, 60455-760

[d]Instituto de Ciências Exatas e da Natureza (ICEN), Universidade da Integração Internacional da Lusofania Afro-Brasileira, Redenção, CE, Brazil, 62790-000

Table S1. Percentage of contribution to RDA achieved for minerals in raw and cooked crab samples

**Elements (%)**	**CRAB 1**				**CRAB 2**				**Industrialized crab meat**			
	In natura		**Cooked**		***In natura***		**Cooked**		***In natura***		**Cooked**	
	**T**	**B**	**T**	**B**	**T**	**B**	**T**	**B**	**T**	**B**	**T**	**B**
Ca	99	13	76	57	124	62	126	50	177	67	121	43
Cr	1074	6.6	1160	31	1877	34	999	14	10000	37	57	0
Cu	590	7.1	580	45	970	65	970	13	200	144	144	100
Fe	37	0	33	0	54	0	34	0	67	5,4	65	4,3
K	42	32	20	21	16	16	13	14	38	1,9	15	0,9
Mg	38	3	34	30	56	64	37	5	59	17	39	21
Mn	5	0.1	5.1	0.7	12	0.5	8.9	0.0	20	0,9	14	2,6
P	65	5.9	64	61	55	1.8	65	3.9	68	33	68	28
Se	608	609	538	345	1667	193	1201	34	255	159	181	73
Zn	210	71	200	45	238	2	219	1	160	30	109	18

T is the total contente and B the bioaccesible content

RDA for Men (31-50 years old)
